# Resistive Switching Memory Devices Based on Body Fluid of *Bombyx mori* L.

**DOI:** 10.3390/mi10080540

**Published:** 2019-08-16

**Authors:** Lu Wang, Dianzhong Wen

**Affiliations:** 1 HLJ Province Key Laboratory of Senior-Education for Electronic Engineering, Heilongjiang University, Harbin 150080, China; 2 Research Center for Fiber Optic Sensing Technology National Local Joint Engineering, Heilongjiang University, Harbin 150080, China

**Keywords:** resistive switching memory devices, *Bombyx mori* body fluid, biomemristor, natural biomaterial

## Abstract

Resistive switching memory devices are strong candidates for next-generation data storage devices. Biological memristors made from renewable natural biomaterials are very promising due to their biocompatibility, biodegradability, and ecological benignity. In this study, a nonvolatile memristor was fabricated using the body fluid of *Bombyx mori* as the dielectric layer. The developed Al/*Bombyx mori* body fluid film/indium tin oxide (ITO) biomemristor exhibited bipolar resistive switching characteristics with a maximum on/off current ratio greater than 10^4^. The device showed a retention time of more than 1 × 10^4^ s without any signs of deterioration, thus proving its good stability and reliability. The resistive switching behavior of the Al/*Bombyx mori* body fluid film/ITO biological memristor is driven by the formation and breakage of conductive filaments formed by the migration of oxygen ions. This study confirms that *Bombyx mori* body fluid, a 100% natural, inexpensive, and abundant material, is a potential candidate as a nonvolatile biomemristor material with broad application prospects.

## 1. Introduction

As the integration and performance of microelectronic chips follow the rapidly increasing trend predicted by Moore’s law, traditional storage technologies based on complementary metal-oxide-semiconductor (CMOS) processes are gradually approaching their physical limits, and the problem of "storage wall" in computer science is becoming increasingly serious and hindering further development. Therefore, there is a need to find new electronic device and storage technologies to enable the development of new computers with larger storage capacities and faster processing speeds. The emergence of resistive memory has given researchers hope for solving these problems. Various dielectric layer materials for resistive memory have been reported [1,2,3,4,5,6,7], mainly including organic molecules and polymers (metal-organic complexes [8], poly(9-vinylcarbazole) (PVK) [9]), oxides [10,11,12] (TiO*_x_* [13,14,15], SiO*_x_* [16,17]), carbon-based materials [16,18,19], and perovskite-type complex oxides [20] (SrTiO_3_ [21,22], BaTiO_4_ [23,24], LaMnO_3_ [25]). These resistive memories have the advantages of low cost, low power consumption, and multistate operation. Resistive memories have become a popular topic of research in the fields of materials science, nanoscience, physics, electronics, and information science.

With the increasing awareness of the need for environmental protection, the use of natural biological materials to develop and produce green, biocompatible, and biodegradable resistive memories has attracted the attention of an increasing number of researchers, and rapid progress has been achieved in this domain in recent years. As previously reported, tungsten/egg albumen/indium tin oxide (ITO)/polyethylene terephthalate devices with egg white as the dielectric layer have an on/off current ratio of approximately 5 × 10^2^ and can be repeatedly cycled for 100 cycles [26]. Similarly, a bipolar resistive switching memristor with egg white as the dielectric layer has an on/off current ratio of 10^2^–10^4^ and can be repeatedly cycled for 120 cycles [27]. For a ferritin-based memristor, the on/off current ratio of the device can be varied from 0 to 5 by tuning the amount of iron in the ferritin [11,28]. A memristor using κ-carrageenan (κ-car) as the resistive switching layer has a low reset voltage (~0.05 V), a high on/off current ratio (>10^3^), and low power consumption [29]. The fabrication of biomemory devices using composite membranes of deoxyribonucleic acid (DNA) and lead sulfide (PbS) nanoparticles has also been reported. Due to the strong electrostatic interaction between the PbS nanoparticles and the phosphate groups of the DNA molecules, such ITO-DNA:PbS-metal devices exhibit bistable memristive effects with high on/off current ratios of close to 4 orders of magnitude [30]. A Ag/leaves/Ti/PET device made from powdered plant leaves has a low on/off current ratio of approximately 30 and a retention time of 10^3^ s, allowing it to function as resistive random access memory (RRAM) [31]. A Ag/cellulose fiber/Al storage device, with an active layer composed of microfibers, has also been fabricated using an electrospinning technique. This device can withstand 6 × 10^3^ cycles, but its on/off current ratio is only approximately 10. The memristive effect arises due to the formation and breakage of Ag conductive filaments in the active cellulose fiber layer [32]. A fibroin protein liquid solution obtained by boiling and degumming cocoons has been used to fabricate an ITO/fibroin/Al memristor. This memristor has a low on/off current ratio of approximately 10 [33].

This paper presents an Al/*Bombyx mori* body fluid film/ITO biomemristor, which was fabricated using a natural biomaterial, namely, *Bombyx mori* body fluid, as the active layer. Experimental results showed that the device exhibited nonvolatile bipolar resistance characteristics, with a maximum on/off current ratio of greater than 10^4^ and a retention time of more than 1 × 10^4^ s, and it could withstand more than 100 write-read-erase-read cycles. The *Bombyx mori* body fluid used in this study can be directly used in the production of biological memristors without any purification, degumming, or other treatment processes. Compared with most other biological memristors, the presented Al/*Bombyx mori* body fluid film/ITO device exhibits higher performance. Moreover, its production process is simpler.

## 2. Materials and Methods

### 2.1. Materials and Service Fabrication

The Al/*Bombyx mori* body fluid film/ITO memristor was manufactured in a clean laboratory. In an ultrasonic cleaner, an ITO/glass substrate was washed successively with deionized water, acetone, and ethanol, each for 15 min. The *Bombyx mori* body fluid was spin-coated onto the ITO/glass substrate (first at 500 rpm for 5 s and then at 4000 rpm for 40 s), and the device was then baked at 105 °C for 10 min. If the drying treatment were to be carried out at a lower temperature, the device performance might be affected due to the high residual water content. Finally, an Al electrode was deposited on the *Bombyx mori* body fluid film via thermal evaporation to serve as the upper electrode of the biomemristor. The single Al electrode had a diameter of 400 μm and a thickness of 220 nm.

### 2.2. Characterization

The microstructure of the *Bombyx mori* body fluid was observed with a transmission electron microscope (TEM) (JEM-2100, JOEL, Tokyo, Japan). A scanning electron microscope (SEM) (Hitachi SU8020, Hitachi, Tokyo, Japan) was used to observe the cross section of the ITO/glass substrate coated with *Bombyx mori* body fluid. The Fourier transform infrared (FT-IR) (PERKIN-ELMER, Waltham, MA, USA) spectrum of the *Bombyx mori* body fluid and the ultraviolet-visible absorption and fluorescence emission spectra of the *Bombyx mori* body fluid film were also measured. Electrochemical analysis was then carried out on the *Bombyx mori* body fluid film using an electrochemical workstation (BAS-100B, BAS, West Lafayette, IN, USA). The electrical properties of the Al/*Bombyx mori* body fluid film/ITO memristor were tested using a semiconductor parametric tester (Keithley 4200, Keithley, Solon, ME, USA). A dynamic signal analyzer (Agilent 35670 A, Agilent, Palo Alto, CA, USA) and a current amplifier circuit were used to test the low-frequency noise of the biomemristor. 

## 3. Results

*Bombyx mori* is an insect that undergoes complete metamorphosis. In its lifetime, it passes through four distinct morphological and functional developmental stages: egg, larva, chrysalis, and moth (adult). The egg stage corresponds to embryogenesis and the development of the larva. The larval stage mainly consists of nutrient intake and growth. The silkworm chrysalis is the stage of metamorphosis from larva to adult. The adult stage is the reproductive stage, in which mating and breeding occur. The *Bombyx mori* body fluid used in this experiment was obtained directly from *Bombyx mori* larvae. This body fluid, which is mainly composed of blood cells and plasma, accounts for 21–25% of the body weight of the *Bombyx mori*.

**Figure 1:**
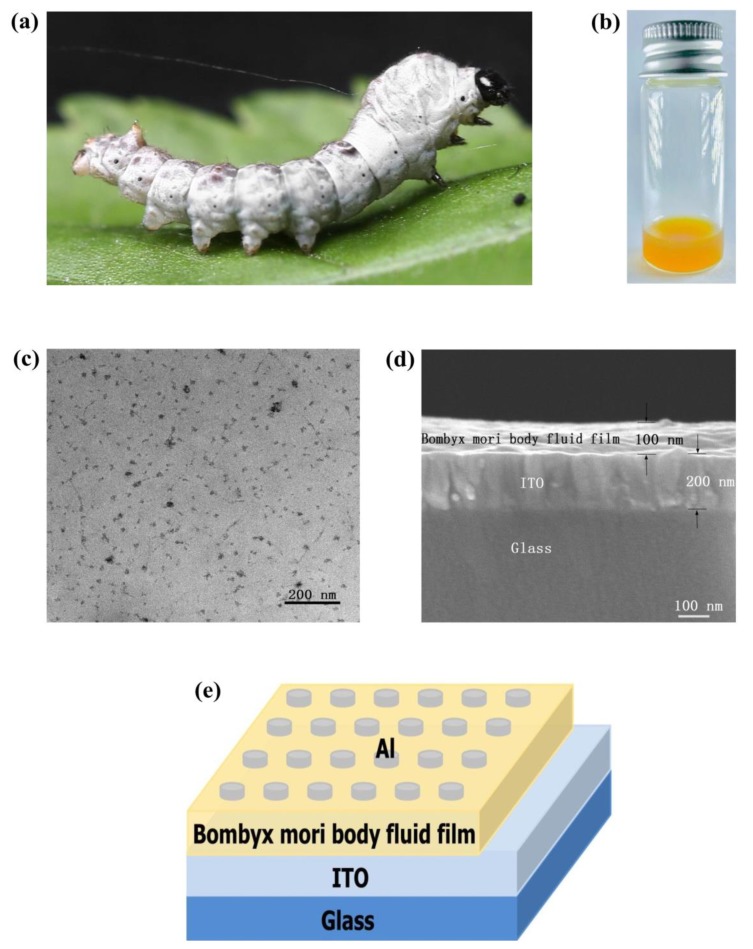
(**a**) Photograph of a *Bombyx mori*; (**b**) Photograph of *Bombyx mori* body fluid; (**c**) TEM image of *Bombyx mori* body fluid; (**d**) SEM image of the cross section of the *Bombyx mori* body fluid film/indium tin oxide (ITO)/glass structure; (**e**) Schematic diagram of the Al/*Bombyx mori* body fluid film/ITO biomemristor.

Figure 1a is a photograph of a *Bombyx mori*, and Figure 1b is a photograph of body fluid directly obtained from a *Bombyx mori*. The microstructure of the *Bombyx mori* body fluid was observed via TEM, as shown in Figure 1c, and the cross section of the body fluid/ITO/glass structure was observed via SEM, as shown in Figure 1d. In the SEM image, the upper layer is a 100 nm film of *Bombyx mori* body fluid, the middle layer is a 200 nm ITO film, and the lower layer is glass. Figure 1e shows the final structure of the Al/*Bombyx mori* body fluid film/ITO biological memristor. From top to bottom, the layers are Al (upper electrode), *Bombyx mori* body fluid film, ITO (lower electrode), and glass.

**Figure 2:**
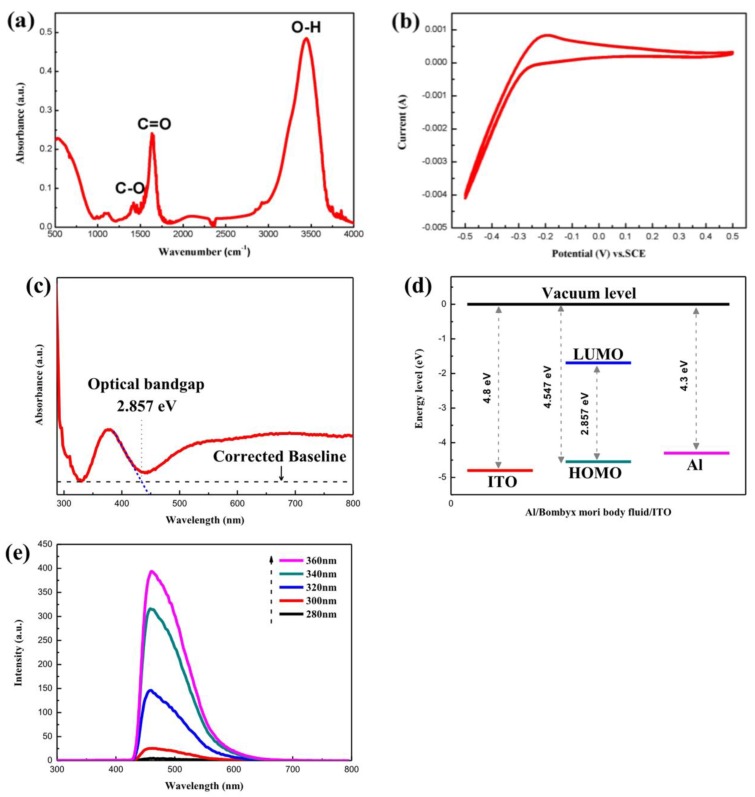
(**a**) Infrared spectrum of *Bombyx mori* body fluid; (**b**) Cyclic voltammetry curves and (**c**) Ultraviolet-visible absorption spectrum of the *Bombyx mori* body fluid film; (**d**) Energy level diagrams of the materials used in the Al/*Bombyx mori* body fluid film/ITO biomemristor; (**e**) Fluorescence emission spectra of the *Bombyx mori* body fluid film at different excitation wavelengths.

The chemical bonds in the *Bombyx mori* body fluid were analyzed via infrared spectroscopy. Figure 2a shows the FT-IR spectrum of the *Bombyx mori* body fluid. The peak at 1418 cm^−1^ was associated with the bending vibrations of carboxylate (C–O) [34]. The peak at 1635 cm^−1^ corresponded to the stretching vibrations of carboxyl groups (C=O) [35]. From 2340 to 3800 cm^−1^, wide peaks associated with the stretching vibrations of hydroxyl groups (OH, ~3445 cm^−1^) [34] could be clearly observed.

The energy levels of the *Bombyx mori* body fluid were analyzed. First, an electrochemical analysis of the *Bombyx mori* body fluid film was performed to determine the highest occupied molecular orbital (HOMO) of the material. The cyclic voltammetry curve of the film obtained in a 0.02 mol/L hydrochloric acid solution is shown in Figure 2b. As seen from this figure, the initial oxidation potential of the saturated calomel electrode relative to the vacuum level (*E_OX_*) was −0.193 V. The HOMO energy level can be obtained via the following formula [36]:

*E_HOMO_* = −4.74 − *E_OX_* (1)

Using this formula, *E_HOMO_* = −4.547 eV could be calculated.

The ultraviolet-visible spectrum of the *Bombyx mori* body fluid film was measured, as shown in Figure 2c. Using this spectrum, the band gap width (*E_g_*) could be determined. *E_g_* is the difference in energy between the HOMO and the lowest unoccupied molecular orbital (LUMO). The wavelength corresponding to *E*_g_ can be calculated from the intersection of the tangent of the starting edge of the peak with the baseline in the ultraviolet-visible absorption spectrum. In this way, a λ value of 434 nm was calculated. According to the relation *Eg* = h*c*/*λ*, the band gap width of the *Bombyx mori* body fluid was thus calculated to be 2.857 eV. Then, the LUMO energy level could be obtained via the following formula [36]:

*E_LUMO_* = *E_HOMO_* + *E_g_* (2)

Using this formula, *E_LUMO_* = −1.690 eV could be calculated. The energy levels of the various materials used in the Al/*Bombyx mori* body fluid film/ITO biomemristor are summarized in Figure 2d.

To further analyze the optical properties of the *Bombyx mori* body fluid film, its fluorescence emission spectra at excitation wavelengths of 280, 300, 320, 340, and 360 nm were measured, as shown in Figure 2e. According to the results, when the excitation wavelength was in the range of 280 to 360 nm, the fluorescence intensity of the *Bombyx mori* body fluid film increased as the excitation wavelength increased.

**Figure 3:**
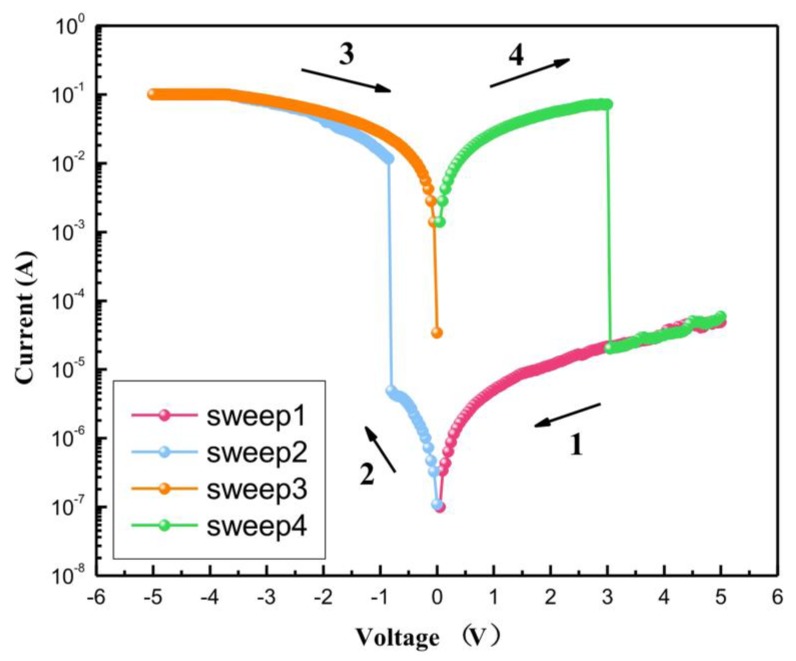
Current-voltage (I-V) characteristics of Al/*Bombyx mori* body fluid film/ITO biomemristor.

The Al/*Bombyx mori* body fluid film/ITO biomemristor exhibited typical bipolar resistive switching characteristics and rewritable flash characteristics. Figure 3 shows a typical SET-RESET cycle for the biomemristor. During this test, a voltage was applied to the Al electrode of the device, and the ITO electrode was grounded. The applied voltage cycle was 5 V → 0 V → −5 V → 0 V → 5 V with a step size of 0.05 V, and the limiting current was set to 0.1 A. When an applied voltage (5 V → 0 V → −5 V) was applied to the Al electrode, the device was initially in a high-resistance state (HRS), that is, the "off state". As the bias voltage decreased, the current also initially showed a steady decrease. When the applied voltage reached −0.85 V (defined as the set voltage, *V_SET_*), the current suddenly increased. The current of the device suddenly changed from 4.87 × 10^−6^ A to 1.15 × 10^−2^ A, that is, the device suddenly changed from an HRS to a low-resistance state (LRS), meaning that the device switched to the "on state". This process is called the SET process. After the device was set, the current varied with the voltage, but the device remained in the LRS. Then, when a voltage cycle of −5 V → 0 V → 5 V was applied to the device, when the voltage reached 3.05 V (defined as the reset voltage, *V_RESET_*), the current suddenly decreased. At this time, the current in the biomemristor suddenly changed from 7.18 × 10^−2^ A to 2.01 × 10^−5^ A, and the device changed from the LRS to the HRS. This process is called the RESET process.

**Figure 4:**
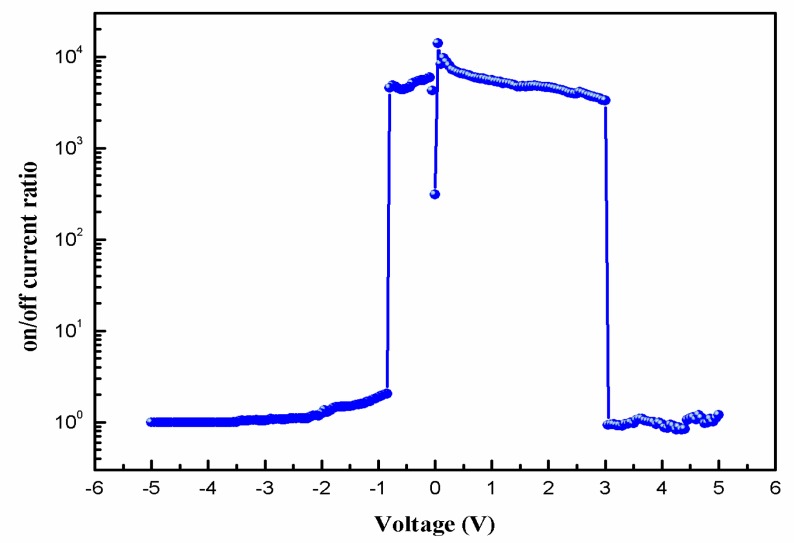
Variation in the on/off current ratio of Al/*Bombyx mori* body fluid film/ITO biomemristor with the applied voltage.

Figure 4 shows the variation in the on/off current ratio of the Al/*Bombyx mori* body fluid film/ITO memristor with the applied voltage. When the voltage was varied from −0.80 to 3.00 V, the on/off current ratio was between 3.34 × 10^3^ and 1.41 × 10^4^. When V = 0.05, the biomemristor had a maximum on/off current ratio of 1.41 × 10^4^. Compared with other biological memristors, the Al/*Bombyx mori* body fluid film/ITO memristor had a relatively high on/off current ratio, indicating that this device has good application prospects as a type of nonvolatile memory.

**Figure 5:**
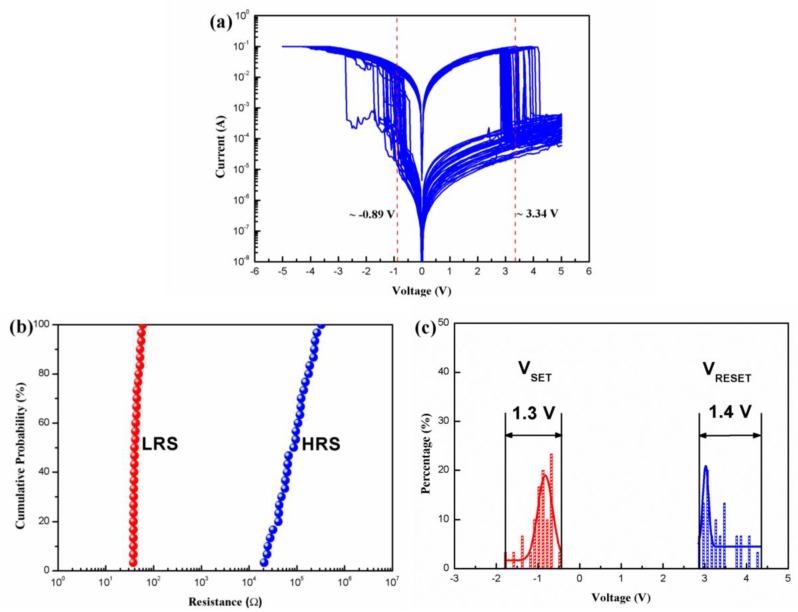
(**a**) Superposition of I-V curves for two biomemristor cells from the same glass substrate measured 15 times for each cell; (**b**) Cumulative probabilities of the resistance of high-resistance state (*R_HRS_*) and resistance of low-resistance state (*R_LRS_*) of the devices; (**c**) *V_SET_* and *V_RESET_* distributions of the biomemristors.

Figure 5a is the superposition I-V curves of two biomemristor cells from the same glass substrate measured 15 times for each cell. It can be seen from this figure that the values of V_SET_ and V_RESET_ changed with cyclic switching. The average of the *V_SET_* for the biomemristor was −0.89 V, whereas that of the *V_RESET_* was 3.34 V. Figure 5b shows the cumulative probabilities of the resistances of HRS (*R_HRS_*) and LRS (*R_LRS_*). *R_LRS_* demonstrated narrow distributions. Figure 5c shows the *V_SET_* and *V_RESET_* distributions of the biomemristors, which were fitted using Gaussian function. Differences between the *V_SET_* and *V_RESET_* of the biomemristors were sufficiently large to ensure that the HRS and LRS of the devices were distinguishable.

**Figure 6:**
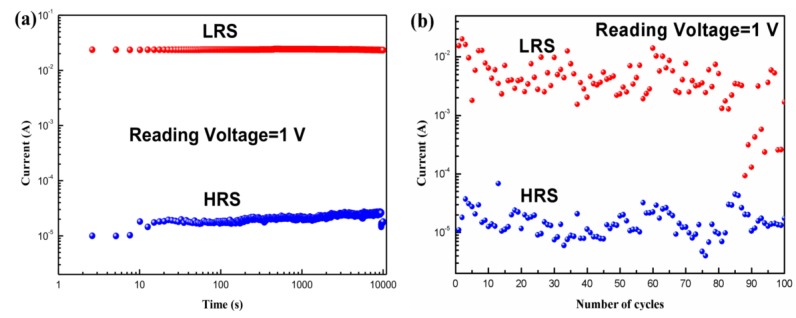
(**a**) Retention properties; (**b**) Endurance properties of Al/*Bombyx mori* body fluid film/ITO biomemristor.

Figure 6a shows the retention properties of the Al/*Bombyx mori* body fluid film/ITO biomemristor. At 1 V, the HRS and LRS of the device remained basically unchanged for 10^4^ s, which means that the device could be stably maintained in either the HRS or the LRS, demonstrating the nonvolatility of the memristor. Figure 6b shows the endurance properties of the biomemristor. The device could be written to and erased by applying pulse signals of 4.5 V/100 ms and −3.5 V/100 ms, respectively. The current of the device was measured at 1 V after each operation. The biomemristor showed rewritable performance over 100 cycles.

**Figure 7:**
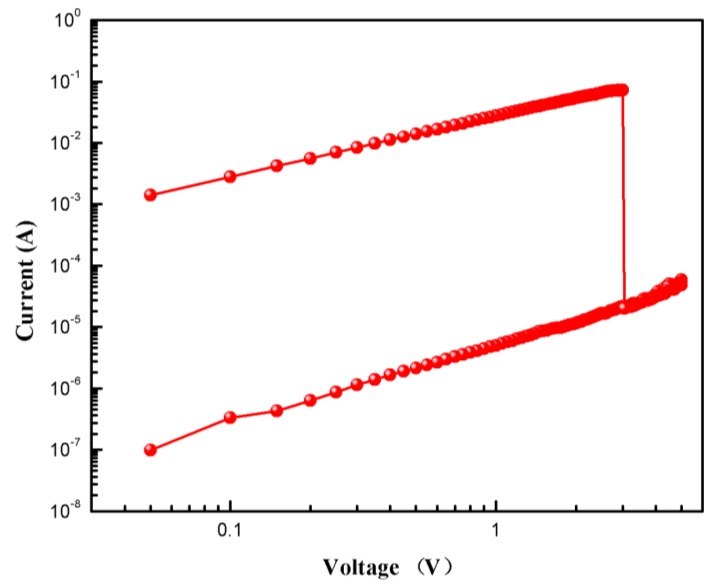
I-V characteristics of Al/*Bombyx mori* body fluid film/ITO biomemristor in double natural logarithmic coordinates.

To study the current transfer mechanism of the biomemristor, we replotted the I-V characteristic curve of the device in double natural logarithmic coordinates, as shown in Figure 7. As can be seen, the slope of the current-voltage characteristic curve was approximately 1 in both the LRS and the HRS, that is, the current and voltage were linear, I∝V, indicating that the conduction current followed Ohm’s law.

**Figure 8:**
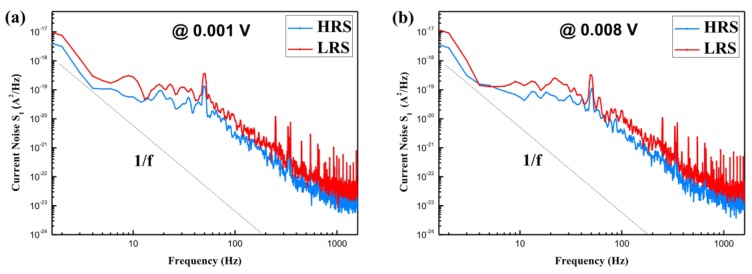
Current noise power spectral density of the Al/*Bombyx mori* body fluid film/ITO biomemristor in the LRS and the HRS under applied voltages of (**a**) 0.001 V and (**b**) 0.008 V.

To study the resistive switching mechanism of the Al/*Bombyx mori* body fluid film/ITO biomemristor, a low-frequency noise power spectral density experiment was carried out. Figure 8 shows the current noise power spectral density of the device for applied voltages of 0.001 and 0.008 V, respectively. It can be seen from this figure that the power spectral density of the current noise in the LRS was higher than that in the HRS. In addition, the power spectral density of the current noise was inversely proportional to the frequency, that is, in the region of the noise spectrum below 1600 Hz, the noise of the device increased sharply as the frequency decreased. The relationship between the noise and the frequency was 1/f. Under different applied voltages, the slope of the power spectral density curve of the current noise was approximately 1 in both the HRS and the LRS, indicating that the noise of the Al/*Bombyx mori* body fluid film/ITO biomemristor was 1/f noise. It can be inferred that, in this biomemristor, the formation and breakage of conductive filaments caused the device to exhibit resistive switching behavior [37].

**Figure 9:**
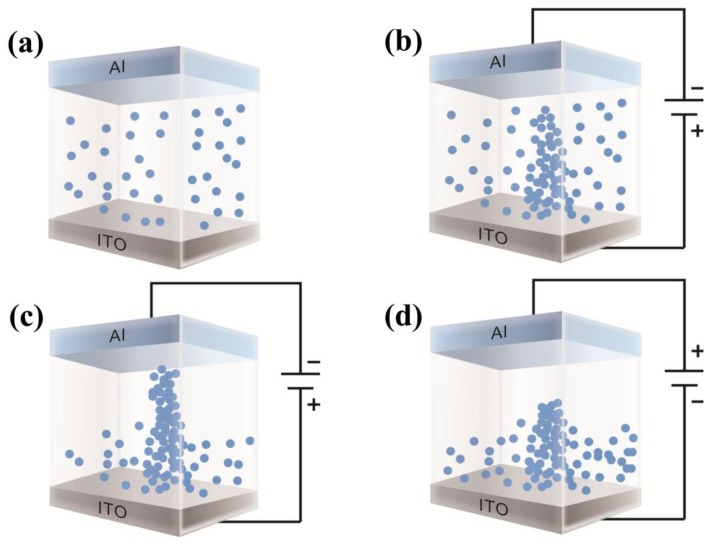
Schematic diagram of the resistive switching mechanism of the Al/*Bombyx mori* body fluid film/ITO biomemristor: (**a**) OFF; (**b**) SET; (**c**) ON; (**d**) RESET.

*Bombyx mori* body fluid is a circulating liquid that bathes all tissues and organs in the body cavity. This fluid contains dry matter in addition to a large amount of water. The main components of the dry matter are proteins, amino acids, other nonprotein nitrogen compounds, trehalose, phosphate, and citrate as well as metal ions, such as sodium, potassium, calcium, and magnesium. It can be seen from the infrared spectrum of the *Bombyx mori* body fluid in Figure 2a that this fluid contains carboxylate (C–O), carboxyl (C=O), and hydroxyl (OH) functional groups. Based on the above analysis, we conclude that the resistive switching properties of this biomemristor mainly originate from the migration of oxygen ions. Accordingly, we have drawn a schematic diagram of the resistive switching mechanism of the developed Al/*Bombyx mori* body fluid film/ITO biomemristor, as shown in Figure 9. When no bias voltage is applied, most oxygen ions in the *Bombyx mori* body fluid film are distributed, as shown in Figure 9a. When a negative voltage is applied to the upper electrode (Al electrode), negatively charged oxygen ions in the *Bombyx mori* body fluid film gradually accumulate on the side closer to the lower electrode (ITO electrode), and they gradually diffuse toward the upper electrode, as shown in Figure 9b. When a sufficiently large negative voltage *V_SET_* is applied, a strong conductive path (which we call a conductive filament) is finally formed, which penetrates the *Bombyx mori* body fluid layer and connects the upper and lower electrodes, as shown in Figure 9c. At this time, the HRS is converted into an LRS, and the device completes the "write" operation. In contrast, when a positive voltage is applied to the upper electrode (Al electrode), the electric charge forming the conductive filament is gradually dissipated. When a sufficiently large positive voltage (*V_RESET_*) is applied, the conductive filament breaks, and the device transitions from the LRS to the HRS, completing the "erase" operation, as shown in Figure 9d.

Therefore, under the action of an electric field, the migration of oxygen ions leads to the formation and breakage of conductive filaments, serving as the resistive switching mechanism of the developed Al/*Bombyx mori* body fluid film/ITO biomemristor.

## 4. Conclusions

In conclusion, we successfully developed an Al/*Bombyx mori* body fluid film/ITO biomemristor, which exhibited nonvolatile resistive switching characteristics with a maximum on/off current ratio greater than 10^4^. The device could store information for more than 1 × 10^4^ s without any deterioration, thus showing its high stability and reliability.

The Al/*Bombyx mori* body fluid film/ITO biomemristor, in which the active layer consists of *Bombyx mori* body fluid, has the advantages of biodegradability, biocompatibility, and environmental friendliness and has broad application prospects in the fields of bioelectronics, information storage, and artificial neural networks. This study is the first to demonstrate that *Bombyx mori* body fluid is a promising natural biomaterial for use in nonvolatile resistive memory, perhaps in future implantable and biocompatible nanoelectronics.
